# Emerging Pharmacotherapeutic Strategies to Overcome Undruggable Proteins in Cancer

**DOI:** 10.7150/ijbs.83026

**Published:** 2023-06-26

**Authors:** Yuqing Lu, Yuewen Yang, Guanghao Zhu, Hairong Zeng, Yiming Fan, Fujia Guo, Dongshu Xu, Boya Wang, Dapeng Chen, Guangbo Ge

**Affiliations:** 1 Dalian Medical University, 116044 Dalian City, Liaoning Province, China.; 2 Shanghai University of Traditional Chinese Medicine, 201203 Shanghai City, China.; 3 Dalian Harmony Medical Testing Laboratory Co., Ltd, 116620 Dalian City, Liaoning Province, China.

**Keywords:** Cancer, Undruggable target, Protein-protein interaction, Intrinsically disordered protein, Targeted protein degradation, Proteolysis targeting chimera

## Abstract

Targeted therapies in cancer treatment can improve *in vivo* efficacy and reduce adverse effects by altering the tissue exposure of specific biomolecules. However, there are still large number of target proteins in cancer are still undruggable, owing to the following factors including (1) lack of ligand-binding pockets, (2) function based on protein-protein interactions (PPIs), (3) the highly specific conserved active sites among protein family members, and (4) the variability of tertiary docking structures. The current status of undruggable targets proteins such as KRAS, TP53, C-MYC, PTP, are carefully introduced in this review. Some novel techniques and drug designing strategies have been applicated for overcoming these undruggable proteins, and the most classic and well-known technology is proteolysis targeting chimeras (PROTACs). In this review, the novel drug development strategies including targeting protein degradation, targeting PPI, targeting intrinsically disordered regions, as well as targeting protein-DNA binding are described, and we also discuss the potential of these strategies for overcoming the undruggable targets. Besides, intelligence-assisted technologies like Alpha-Fold help us a lot to predict the protein structure, which is beneficial for drug development. The discovery of new targets and the development of drugs targeting them, especially those undruggable targets, remain a huge challenge. New drug development strategies, better extraction processes that do not disrupt protein-protein interactions, and more precise artificial intelligence technologies may provide significant assistance in overcoming these undruggable targets.

## 1. Introduction

The incidence of cancer and associated mortality rates continue to increase globally. Cancer is the leading cause of death before the age of 70 years in 112 countries and either the third or fourth in 23 others. Cancer accounted for approximately 19.3 million new cases and nearly 10 million deaths worldwide in 2020 and 28.4 million new cases are anticipated in 2040^1^. At present, surgery, radiation therapy, and chemotherapy are the main strategies for cancer^2^. The success of surgical treatment is usually dependent on the absence of distant metastasis and local infiltration of tumor cells. Chemotherapy regimens use cytotoxic agents to prevent the proliferation, invasion, and metastasis of cancer cells. In 1949, the nitrogen mustard alkylating agent mechlorethamine was marketed as the first anti-cancer drug. Since then, the number of anti-cancer drugs entering the market has gradually increased^3^. Oncology remains the leading indication for Food and Drug Administration (FDA)-approved drugs in the past 2022^4^. Unfortunately, many promising and experimentally validated cancer targets are not within the scope of drug modifiability, such as the transcription factors (TFs). Besides cancer, in other diseases such as autoimmune diseases, neurodegenerative diseases, there are also some identified undruggable targets, and these identified targets are of great interest e.g., STAT3, lymphoid-specific tyrosine phosphatase, tau, alpha-synuclein, etc. Even though autoimmune diseases are considered rare compared to cancer, there are nearly 100 different autoimmune diseases that affect an additional 3% of the population^5^. Neurodegenerative diseases are diseases characterized by progressive impairment of motor and/or cognitive functions^6^. The number of cases worldwide is growing rapidly, especially in the context of the general trend of aging populations worldwide. However, their drug development is currently facing challenges 7, 8.

**Figure 1:**
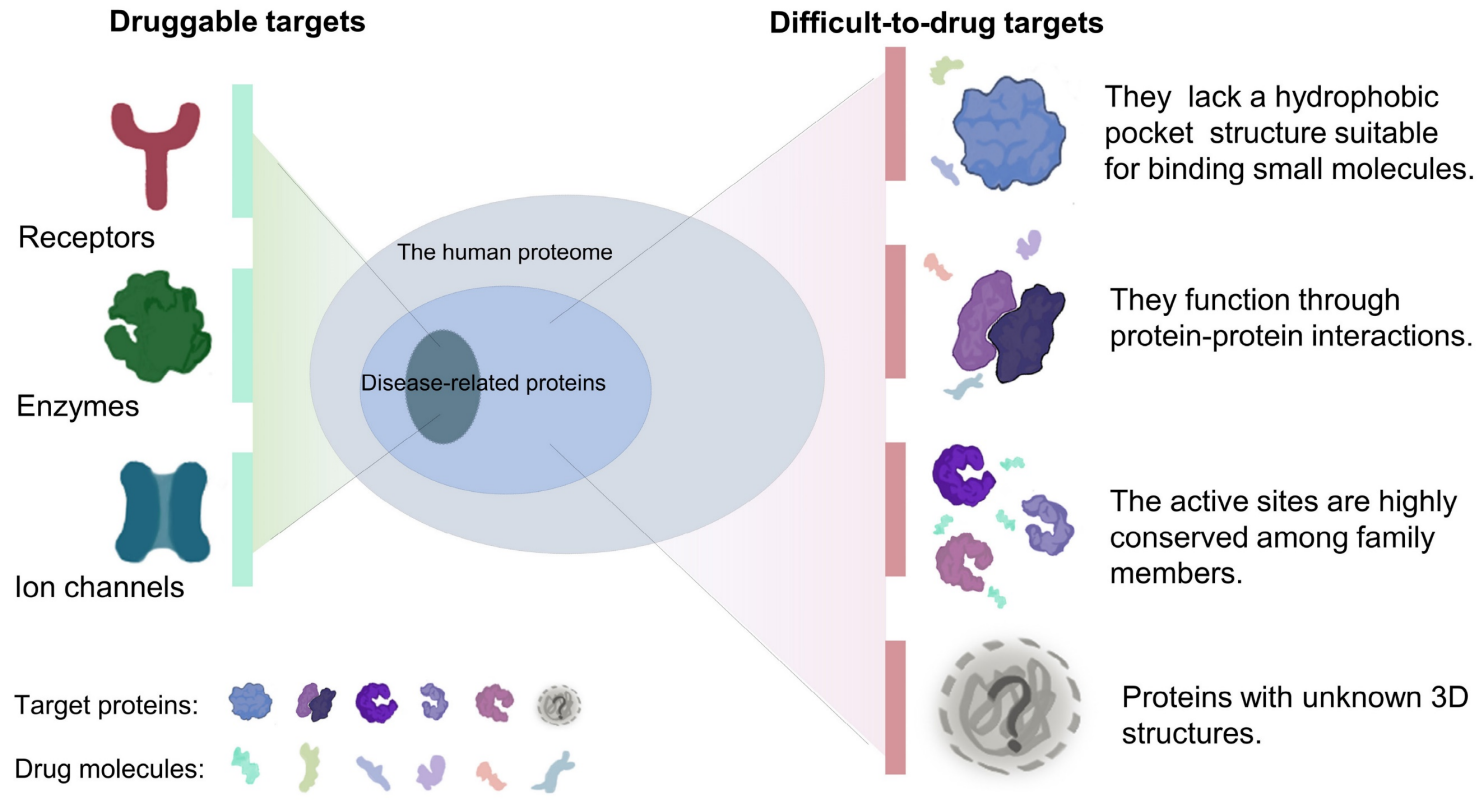
In fact, a few cancer-related targets are druggable, most are difficult to target, and often share some common characteristics.

The therapeutic effects of drugs are traditionally determined by comparison of the efficacy and adverse reactions. Target-based drugs have gradually replaced traditional therapeutics as technologies have developed into the 21^st^ century 9, 10. Most targeted therapies aim at a specific ligand or modulates the function of a target protein. Cancers are usually caused by malfunction of various proteins, but only a relatively small fraction of these proteins can be targeted by small-molecule drugs or biologics^11^. Compared with traditional small molecule drugs, biologics, mainly monoclonal antibodies, have higher specificity and affinity^12^, ^13^. However, due to large molecular weights, most of biologics can only act on extracellular targets^13^-^15^. Nonetheless, a subset of targets is difficult to access by both small-molecule drugs and biologics. It is estimated that only 15% of drug targets (including enzymes, ion channels, and receptors) are considered druggable, while the remaining 85% are considered undruggable^16^. These so-called undruggable proteins tend to have several characteristics, including: (1) the lack of a hydrophobic pocket structure suitable for binding small molecules; (2) function *via* protein-protein interactions and formation of protein complexes; (3) highly conserved active sites that respond to specific inhibitors; and (4) intrinsically disordered or unknown tertiary structures (Figure 1). Based on the function of the target proteins in cancer, these undruggable targets can be classified as follows: (1) RAS family proteins. RAS proteins are small guanosine triphosphatases (GTPases) that play an important role in cell signaling transduction 17. RAS is one of the most widely studied targets in cancer, and its mutations have a definite pathogenic role in many cancers, such as lung cancer and pancreatic cancer^18^, ^19^. The current status of its drug development has been dramatically transformed by relentless efforts. (2) Transcription factors, such as p53. Proteins involved in transcription mediate the expression of abnormal genes that lead to tumorigenesis and progression^20^. p53 is one of the mutated tumor suppressors in cancer. (3) Proteins involved in epigenetic regulation, such as protein tyrosine phosphatases (PTPs). PTPs and kinases together regulate tyrosine phosphorylation of proteins^21^, and their family members play a dual role in carcinogenesis^22^.

Currently, researches try hard to overcome these undruggable targets owing to the new technology. With the continued advancements in drug development, various proteins are validated as potential drug targets. Most approaches to targeting proteins act by modulating the specific activity of the target protein, such as enzyme inhibition or ligand blocking. However, undruggable targets usually do not have enzymatic activity and an obvious active site. In such cases, a direct approach by selective degradation of the target protein is promising. TFs exert their biological activity through PPI and protein-DNA binding, thus facilitating the recruitment of other effectors to perform different functions, which are directly or indirectly involved in a variety of cancer-related gene expression and transcriptional abnormalities. Therefore, by blocking PPIs or protein-DNA interactions is another approach. In this process, the intervention of computer-aided drug development (CADD) and AI greatly facilitates the process of drug discovery and development. The purpose of this review is to provide an overview of the characteristics of undruggable targets in cancer. The drug development strategies to overcome the undruggable targets and current challenges in targeted therapies were also summarized. There are many undruggable targets in cancer^11^, ^23^, ^24^, and the widely studied targets that have great therapeutic potential were selected as representative targets to introduce.

## 2. Undruggable targets in cancer

### 2.1. RAS proteins as “switches” for signal transduction

Mutations to RAS proteins are considered genetic drivers of multiple cancer types, and drug development is challenging. This is mainly because of the lack of pharmacologically actionable pockets for binding of small-molecule drugs. Further, drug development is complicated by the affinity of RAS proteins for guanosine-5'-triphosphate (GTP) combined with high intracellular GTP concentrations 25. RAS proteins are small GTPases that regulate cell growth, differentiation, and apoptosis. There are three isoforms of Ras proteins: K-RAS, N-RAS, and H-RAS^26^. RAS mutations are among the most common drivers of human cancers 27. K-RAS is the most mutated oncogene, occurring in 27%-39%, 40%-54%, and 86%-96% of lung, colorectal, and pancreatic cancers, respectively^28^, ^29^. Missense mutations are reported to increase the affinity of RAS proteins to GTP and lower the enzymatic activity of GTPase-activating proteins. This would leave KRAS in the “on” state and dysregulation of various signaling pathways that are dependent on active RAS regulation^30^-^32^.

In the past decades, attempts to overcome KRAS have been made from different perspectives. More attention has been paid to indirect approaches, including (1) targeting upstream molecules; (2) targeting downstream effector molecules; (3) interfering with RAS mRNA expression; (4) targeting different metabolic processes associated with RAS mutations; and (5) screening of lethal synthetic interactors^17^. Small-molecule inhibitors (SMIs) that block RAS translocation to the cell membrane by targeting upstream molecules, such as PDEδ, SHP2, and STK19, include NHTD 33, JAB-3068 (ClinicalTrials.gov identifier: NCT03565003), and ZT-12-037-01 34, among others. Inhibitors that block downstream adverse events by targeting downstream molecules, such as the RAF-MEK-ERK cascade and PI3K-AKT-mTOR signaling pathway, include RO5126766^35^, BVD523^36^, and NVP-BEZ235^37^. Inhibitors that impede the growth and metabolism of tumor cells by targeting metabolic processes associated with RAS mutations, such as autophagy, may have anti-tumor activities in combination with the above inhibitors^38^. Small interfering RNA exhibits anti-proliferative effects by interfering with RAS expression in tumors through delivery mediators^39^, ^40^. These methods have catalyzed many inhibitor molecules. However, many inhibitors have been difficult to translate to clinic applications due to safety concerns, limited anti-tumor activities, and other issues. In addition to SMIs, attempts have been made to develop degraders of KRAS. As G12C covalent inhibitors, ARS-1620 and Adagrasib were linked to E3 ligase to develop PROTACs. Experiments showed that KRAS^G12C^ degraders showed blockade of downstream signaling. In addition, they effectively inhibited the proliferation of tumor cells^41^, ^42^. Targeting protein degradation is a novel pathway to develop KRAS inhibitors.

Although drugs that directly target the RAS are considered elusive, the discovery of a new allosteric site in KRAS (G12C) has shown new hope for targeting KRAS. AMG510 (Sotorasib) and MRTX849 (Adagrasib) were recently approved for the treatment of non-small cell lung cancer (NSCLC) carrying the KRASG12C mutation^43^, indicating that KRAS is no longer considered clinically undruggable. The KRAS^G12C^ mutation occurs in about 13% of lung adenocarcinomas and about 3% of colorectal adenocarcinomas^44^, ^45^, and this mutant has a cysteine residue (glycine in position 12 becomes cysteine) that is used to design covalent inhibitors^46^. In cancer cells, KRAS ^G12C^ has been shown to cycle rapidly between an active GTP-bound state and an inactive GDP-bound state^47^. AMG510 is a selective inhibitor that binds to the cysteine residue in the Switch Ⅱ pocket of KRAS^G12C^ and irreversibly locks KRAS in an inactive state. Its binding and potency are enhanced by binding to a novel His groove on KRAS compared to previous preclinical attempts^44^. In the clinical phase I/II trial (ClinicalTrials.gov identifier: NCT03600883), AMG510 led to KRAS^G12C^ tumor regression and demonstrated a favorable safety profile. Treatment-related adverse effects included diarrhea, vomiting, elevated alanine aminotransferase levels, and elevated aspartate aminotransferase^48^. The phase II trial was evaluated in 126 patients with KRAS^G12C^ mutant NSCLC and the results were positive. Their objective response rate and disease control rate were 37.1% and 80.6%, respectively, with median remission duration and median progression-free survival of 10 months and 6.8 months, respectively^49^. The excellent trial results led to the accelerated approval of AMG510 by the FDA. Several clinical trials related to AMG510 combination therapy are still ongoing (Table 1). As the development of KRAS^G12C^-targeted therapeutics has progressed at a rapid pace, there have been some notable achievements in the development of drugs for KRAS^G12D^. In preclinical trials, MRTX1133, a KRAS^G12D^ inhibitor, mediated the apparent regression of KRAS^G12D^ mutant pancreatic ductal adenocarcinoma^50^-^52^. Currently, MRTX1133 is in a clinical trial to evaluate its safety, tolerability, and antitumor activity in patients with advanced solid tumor malignancies harboring KRAS^G12D^ mutations (ClinicalTrials.gov Identifier: NCT05737706).

However, acquired resistance ultimately occurs in most patients treated with monotherapy, and KRAS^G12C^ inhibitors do not appear to be an exception. Several possible mechanisms have been proposed to explain this phenomenon, including acquired mutations in KRAS^53^, ^54^, activation of associated parallel signaling pathways^53^, and phenotypic transformation^55^. It has been shown that KRAS^G12C^ inhibitors will lead to multiple different KRAS secondary mutations that can interfere with drug binding and have drug heterogeneity^56^. Bypass mechanisms of drug resistance include activation of upstream regulators, such as EGFR, MET, and RET^57^, ^58^; activation of downstream effectors, such as MEK and RAF^53^, ^59^; and activation of wild-type RAS 60. Epithelial mesenchymal transition is also a mechanism of drug resistance^53^. It modulates and activates the PI3K pathway via the IGFR-IRS1 pathway, leading to endogenous and acquired drug resistance^61^. The mechanisms of acquired resistance to KRAS^G12C^ inhibitors are complex, and different mechanisms can coexist in the same patient. Combination therapy is an important approach to prevent or delay the onset of resistance, and many combination regimens are currently in clinical trials (Table 1), but attention needs to be paid to the occurrence of adverse events.

### 2.2. Undruggable proteins involved in transcription

TP53 is an important tumor suppressor gene that is mutated in more than half of all human cancers. However, drugs that act directly on this target are difficult to develop because p53 lacks deep pockets for binding SMIs, lacks enzymatic activity, and is in the nucleus. The p53 protein is a typical TF. Over the past three decades, the understanding of p53 has undergone three shifts: from an oncoprotein antigen, to an oncogene, to the “Guardian of the Genome” 62. Activation of wild-type p53 in response to cellular damage caused by stressors, such as hypoxia and DNA damage, promotes cell cycle arrest, apoptosis, or senescence, thereby avoiding cellular carcinogenesis^63^-^66^. Therefore, inactivation of normal p53 function in cells often leads to carcinogenesis, and genetic mutation is the main mechanism underlying inactivation of p53. This leads to four possible consequences: loss of function, gain of function, dominant negative effects, or no effect on normal function^67^. Thus, mutated p53 may not only lose normal oncogenic function, but may also exhibit dominant negative effects and/or gain of function, thus acting as a cancer promoter. Although degradation of mutant p53 can be induced^68^ or function can be restored with arsenic trioxide^69^, the existence of different types of p53 mutants can inhibit application of this approach. In addition, various inhibitors of histone deacetylase 6 and HSP90 have been found to induce degradation of mutant p53 70-72. Specific deoxyribonucleases designed to target the mutation site of p53 can degrade mutant p53 transcripts^73^, ^74^, thereby reducing protein expression. Drug development for wild-type p53 has focused on interfering with ubiquitination of p53 by MDM2, thereby stabilizing the p53 protein^75^, ^76^. To date, several small-molecule and natural drugs, such as nutlin-3a^77^, ^78^, have been developed to inhibit interactions between p53 and MDM2.

Upregulation of MYC expression frequently occurs in cancers. A recent analysis of more than 9000 human cancers showed that MYC gene amplification occurs in approximately 28% of malignancies^79^. However, the design of MYC inhibitors is limited due to: (1) intrinsically disordered, but functionally important, domains and lack of enzymatically active sites; (2) high affinity interactions with MAX; (3) partial functional redundancy of family members; and (4) location mainly in the nucleus^80^. C-MYC, L-MYC, and N-MYC are TFs that encode MYC oncoproteins, which are known as “super” TFs that regulate the expression of genes involved in a variety of cellular processes^81^-^83^. Under normal conditions, the expression levels of C-MYC, L-MYC, and N-MYC are strictly limited by various mechanisms^84^-^86^, but are often dysregulated in human cancers. Insertion of a retroviral promoter and chromosomal translocation/amplification can induce MYC overexpression^82^. Overall, N-MYC and L-MYC are reportedly amplified in less than 7% of cancers^79^, whereas C-MYC is amplified in 21%^87^. Notably, even transient inactivation of MYC leads to tumor regression^88^-^90^, suggesting that the modulation of oncogenic MYC is a feasible strategy for cancer treatment. However, the development of drugs directly targeting MYC has been challenging due to the lack of a specific active site for binding of small molecules and location, which is primarily in the nucleus. Therefore, compounds directly targeting MYC have not yet been tested in clinical trials. For these reasons, indirect methods to inhibit the oncogenic function of MYC, such as targeting transcription, translation, or the MYC-MAX complex, have been intensively investigated. The most extensively studied compound directly targeting MYC is Omomyc^91^, which can potentially damage the MYC/MAX/MXD network and has been demonstrated to trigger tumor regression in a variety of cancer models 92-99. Furthermore, the results of recent animal experiments suggest that the adverse side effects induced by the use of Omomyc are mild and reversible, and the therapeutic effect is improved in combination with paclitaxel 94. Recently, GT19715, a novel dual C-MYC/GSPT1 degrader, was reported to effectively degrade C-MYC protein both in vivo and in vitro and to inhibit tumor growth at low doses in Acute Myeloid Leukemia and Lymphomas^100^. Although no therapies targeting MYC have been approved for clinical use, research conducted over the past 20 years has provided a solid foundation for the study of MYC-targeted inhibitors.

As a TF, STAT3 plays significant roles in a wide variety of biological processes. However, current drug development targeting STAT3 is limited due to: (1) the highly homologous SH2 structural domain shared by STAT family members^101^; and (2) the transcriptional activity of the monomeric STAT3 protein, which partially blocks the activities of inhibitors targeting the SH2 structural domain to prevent the formation of STAT3 dimers^102^. As a cytoplasmic TF, STAT3 modulates cell differentiation, proliferation, and apoptosis, in addition to angiogenesis, inflammation, and immune responses^103^. Among the seven conserved STAT family members, tumor cells often overexpress STAT3, which plays an essential role in antitumor immunity 104, 105. STAT3 is a meeting point for a number of oncogenic pathways and is constitutively activated in both tumor cells and tumor-infiltrating immune cells. Overexpression of STAT3 impedes the antitumor immune response by inhibiting expression of mediators necessary for activation of the immune response against tumor cells^106^, ^107^. Besides, STAT3 can induce differentiation and proliferation of Th17 cells by enhancing expression of RAR-related orphan receptor gamma. It also suppresses the initial differentiation of regulatory T cells (Tregs) by suppressing expression of forkhead box P3, which plays vital roles in various autoimmune diseases^108^. Inhibition of STAT3 promotes the growth and differentiation of Tregs and regulates the balance of Tregs and Th17 cells, which can improve symptoms of autoimmune diseases^109^-^111^. Drug development against STAT3 can be broadly divided into two types: one targeting upstream molecules and the other directly targeting STAT3. Direct targeting involves inhibition of STAT3 phosphorylation, dimerization, nuclear translocation, and binding to DNA. Blocking of upstream molecules, such as JAK, can inhibit a variety of downstream pathways, resulting in undesirable consequences^112^, ^113^. Among the direct targeting strategies, the SH2 structural domain has been widely studied because of the key role in STAT3 activation. However, this strategy is limited because targeting the SH2 structural domain does not completely inhibit STAT3^102^. In recent years, degraders have become the focus of attention in drug development. Prof. Shaomeng Wang's team developed the small molecule SD-36, which is a selective degrader of STAT3^114^. In leukemia cell lines and lymphoma cell lines, SD-36 efficiently and selectively reduced STAT3 levels. In a mouse Molm-16 xenograft model, it achieved significant degradation of STAT3 and complete and durable tumor regression^114^. However, the development of PROTACs seems to be more challenging. To date, a number of STAT3 inhibitors have been investigated in clinical trials, but none are currently approved for clinical use, which has led to the development of more effective STAT3 inhibitors and further exploration of additional drug development strategies.

Variable splicing of pre-mRNA of the androgen receptor (AR) causes resistance of the AR-V7 splicing variant to AR signaling inhibitor therapy. In addition, the AR-V7 is considered difficult to target due to the lack of a ligand-binding domain for androgen and antagonists. AR signaling plays a non-negligible role in the development of prostate cancer^115^. As the mainstay treatment for prostate cancer, androgen deprivation therapy works by limiting the availability of androgens^116^. However, tumor cells are known to develop adaptive resistance to almost all targeted therapies and long-term treatment can eventually lead to castration-resistant prostate cancer (CRPC)^117^-^119^. The mechanism underlying treatment resistance is related to reactivation of AR signaling and formation of the AR-V7 splicing variant^120^, ^121^. In prostate cancer, cryptic exon 3 of intron 3 of the AR pre-mRNA sequence is selected by the spliceosome to replace the subsequent AR exon, resulting in the AR-V7 splicing variant^122^, ^123^. This in turn forms a heterodimer that can activate downstream target genes in the absence of androgens. Hence, next-generation drugs that directly or indirectly target AR-V7 signaling are urgently needed.

β-catenin is a classical oncogenic TF that is a key effector involved in the Wnt oncogenic pathway. Drug development targeting β-catenin is challenging due to: (1) the lack of deep pockets for binding SMIs; and (2) the tendency of β-catenin to bind to TCF-4 with low affinity, although the interaction surface is relatively large^124^. β-catenin is a multifunctional protein and a key transducer of the classical Wnt signaling pathway, which participates in the regulation of cell differentiation and proliferation 125. Without Wnt ligands, β-catenin is recruited to a disruption complex composed of APC and AXIN that promotes phosphorylation of β-catenin, leading to ubiquitination and proteasomal degradation to maintain low expression levels in the cytoplasm^126^. When Wnt is activated or mutated, unphosphorylated β-catenin accumulates in the cytoplasm and subsequently migrates to the nucleus to interact with TCF/LEF and coactivators, resulting in transcription of specific target genes encoding oncoproteins^127^, ^128^. Therefore, targeting β-catenin presents a very attractive anticancer treatment strategy. However, β-catenin is rarely targeted^124^ and no inhibitors targeting β-catenin to inhibit the Wnt signaling pathway have emerged.

Although HOXA9 and MEIS1 play synergistic and pathogenic roles in acute myeloid leukemia (AML), both molecules are considered difficult to drug due to the lack of deep pockets for binding SMIs. HOXA9 is a member of the HOX gene family of homologous TFs. AML is the most extensively studied disease involving dysregulation of HOX gene expression, as HOXA9 is overexpressed in about 50%^129^. Hence, HOXA9 is a potential target for the treatment of AML. Hematopoietic stem and progenitor cells normally express high levels of HOXA9, although expression levels are relatively decreased in mature cells^130^. Aberrant expression of HOXA9 is a salient feature of AML driven by multiple oncogenes. In current drug development, HOXA9 expression is often downregulated by indirect methods and thus is considered an undruggable target. Dysregulation of HOXA9 often occurs in conjunction with upstream genetic alterations, such as mixed-lineage leukemia (MLL) fusions, NUP98 fusions, nuclear translocation of phospholipid 1, and overexpression of CDX2, all of which can upregulate the expression of HOXA9^131^, ^132^. Many studies have proposed regulation of HOXA9 expression by targeting MLL fusion-related proteins, such as DOT1L and Menin^133^. MEIS1 is a member of the MEIS subfamily of TFs and plays important roles in leukemia and many solid tumors^134^. MEIS1 and HOXA9 act together to accelerate leukemogenesis by promoting cell proliferation and inhibiting apoptosis^134^, ^135^. In terms of drug development, like HOXA9, most strategies have focused on targeting MLL fusion-related proteins.

As an oncogenic TF, the EWS-FLI1 chimeric fusion protein is an attractive therapeutic target for Ewing sarcoma (ES). Although not present in normal cells, EWS-FLI1 is considered an intrinsically disordered protein (IDP) that is difficult to directly target^136^. ES is a malignant tumor of the bone and soft tissue and is the second most common primary bone malignancy in pediatric patients 137, 138. EWS-FLI1, a major regulator of ES, is a clear target for the treatment of ES, as successful inhibition has led to tumor regression^139^, ^140^. As a potential therapeutic target, there are no drugs that act directly on the EWS-FLI1 fusion protein due to the lack of stable structures and enzymatic activities.

SMARCA2 is an ATPase subunit of the Switch/Sucrose Non-Fermentable chromatin remodeling complex. It is highly homologous to another subunit, SMARCA4, and together they regulate the repair of damaged DNA and DNA transcription^141^. In many cancers, especially NSCLC, SMARCA4 mutations result in expression deficiency^142^. Studies have shown a synthetic lethal effect of SMARCA2 with SMARCA4^143^. Synthetic lethality has been described as the interaction of two genes, where when one is repressed, the other can functionally compensate or replace the function of the first, while the loss of function of both is lethal to the cell, offering the possibility of indirect targeting of non-drug acting targets. Therefore, the strategy of targeting SMARCA2 to treat cancers with SMARCA4 mutations has attracted a lot of attention. However, due to the high similarity of SMARCA2 and SMARCA4 proteins, the selection of inhibitors is difficult to develop.

### 2.3. Undruggable proteins involved in epigenetic regulation

PTPs are considered difficult to target due to (1) the highly conserved active site of family members, (2) the active site of PTP is positively charged, thus screening against the active site often yields negatively charged phosphate analogs, in addition to poor cell permeability and pharmacokinetic properties, and (3) the side chains of catalytic cysteines that act as sulfate anions in the positively charged active site are major targets of various electrophiles, which can interfere with high-throughput screening (HTS)^144^. Tyrosine phosphorylation of intracellular proteins is regulated by the antagonistic activities of protein tyrosine kinases and PTPs, which remove phosphate groups from proteins by hydrolysis^21^, ^145^. Numerous studies have shown that disruption of tyrosine phosphorylation caused by dysregulation of PTP expression is involved in the pathogenesis of various cancers, autoimmune diseases, and diabetes^146^. The human genome encodes more than 100 PTPs^147^, which are classified as a superfamily characterized by a conserved CX5R motif at the active site^148^. Numerous studies have shown that members of the PTP family play dual roles in oncogenesis and can therefore be classified as tumor suppressors or oncogenic PTPs^22^. In addition, PTPs are also involved in progression of autoimmune diseases. The single-nucleotide polymorphism c.1858C>T (rs2476601) of PTPN22, which encodes protein tyrosine phosphatase N22, is related to a variety of autoimmune diseases 149. Although the roles of many PTPs have been well-documented in various diseases 150, no drugs targeting PTPs have yet been approved for clinical use.

Several drugs currently being tested in clinical and preclinical trials target epigenetic regulatory proteins, such as DNA methyltransferases and histone deacetylases, among others. In contrast, studies of histone acetyltransferases (HATs) as potential inhibitors are challenged by several factors, including: (1) the variety of cellular substrates ranging from histones and TFs to enzymes and nuclear receptors; and (2) the formation of multiprotein complexes that determine function, enzymatic activity, and substrate specificity^151^, ^152^, which limit translation to cellular and *in vivo* experiments. Epigenetic modifications do not alter linear DNA sequences, but directly affect DNA conformation and gene activation or repression, and therefore have great therapeutic potential for treatment of human diseases^153^. Epigenetic regulatory proteins include a broad group of “writers,” “readers,” and “erasers,” which have distinctly different functions^154^. HATs are classified as “readers” that facilitate acetylation of lysine residues of cellular proteins. As compared to other family members, CBP/p300 has been more intensively studied in the field of cancer. It is unclear why both CBP/p300 deletion and overexpression can promote tumorigenesis^155^. However, several studies have shown that CBP/p300 inhibitors impede cancer cell survival, proliferation, and metastasis in a variety of cancer types^156^-^159^. HAT inhibitors are currently classified as dual-substrate inhibitors, natural product inhibitors, and synthetic SMIs^160^. Two CBP/p300 inhibitors are currently being tested in clinical trials as potential targets for treatment of cancer patients: CCS1477 (ClinicalTrials.gov identifier: NCT03568656) and FT-7051 (ClinicalTrials.gov identifier: NCT04575766). In addition, EP31670, a dual BET and CBP/p300 inhibitor, was recently approved for a phase 1 study of patients with advanced solid tumors (ClinicalTrials.gov identifier: NCT05488548). HATs were among the first epigenetic modifiers to be identified, but still no potent, selective drugs have been approved for clinical use due to the tertiary structures (Table 2).

As mentioned above, many promising targets are facing difficulties in drug development. The following sections describe recent technological advances that have facilitated undruggable targets as promising therapeutics for treatment of different cancers.

## 3.Targeted protein degradation

The human body has a series of sophisticated systems, such as the ubiquitin-proteasome system (UPS) and the lysosomal system, that maintain protein homeostasis. TPD technology takes advantage of this natural mechanism and is able to directly degrade target proteins at the post-translational level with high selectivity and efficiency.

### 3.1. PROTACs

The term PROTAC was introduced in 2001 to describe a small bifunctional molecule. It can bind to both target proteins and E3 ubiquitin ligases, which leads to the ubiquitination and degradation of target proteins, and can catalyze the degradation of multiple target proteins. Following the synthesis of PROTAC-1 containing IκBα phosphopeptide and ovalicin^161^, other small-molecule PROTACs have emerged based on MDM2 E3 ligase^162^, IAP1 E3 ligase^163^, VHL, and CRBN^164^, ^165^. In addition, Kelch-like ECH-associated protein 1-based PROTACs have emerged, including peptide-based and small molecule degraders^166^, ^167^. An increasing number of proteins are proving to be targets of PROTACs, AR, ER, STAT3, etc. As of 2019, two PROTACs have been tested in clinical trials conducted in the U.S. for the treatment of refractory prostate and breast cancers (ClinicalTrials.gov identifiers: NCT03888612 and NCT04072952). Currently, there are at least 20 PROTACs in clinical trials and the number will continue to increase. As compared to conventional drugs, the advantages of PROTACs include: (1) they can disrupt multiple functions of proteins; (2) they have more complete and longer-lasting therapeutic effects; (3) they require relatively lower affinity; and (4) they can prevent the development of adaptive drug resistance^168^. PROTACs use the cellular protein degradation machinery (i.e., UPS) to remove specific target proteins and thus have great potential for targeting undruggable proteins^101^ (Table 3).

ARV-110 is an oral PROTAC developed by Arvinas that selectively targets and degrades AR and is proposed to be developed for the treatment of metastatic castration-resistant prostate cancer 169. In populations with specific genetic mutations, ARV-110 reduced prostate-specific antigen levels by more than 50% in 40% of patients with metastatic desmoplastic resistant prostate cancer^169^. Based on safety, pharmacokinetics and efficacy, 420 mg QD was selected as the recommended clinical phase 2 dose (RP2D). Among the 113 subjects treated with RP2D, there were no grade ≥4 tx-related adverse events, of which nausea, vomiting, fatigue, and decreased appetite were common^170^. ARV-471 is a protein degrader that targets the estrogen receptor (ER) and is proposed for the treatment of ER+/HER2- locally advanced or metastatic breast cancer. Phase I clinical data for ARV-471 also showed that high levels of ER degradation (89%) were observed at dose levels of 30-700 mg with good safety and tolerability^171^ In the phase Ⅱ clinical trial, 100% of 35 patients were previously treated with CDK4/6 inhibitors, 74% with fulvestrant, and 74% with chemotherapy. the clinical benefit rate (CBR) for ARV-471 200 mg QD was 37.1%, and the CBR for evaluable patients with mutant ESR1 (n=19) was 47.4%, and substantial on-treatment reductions in mutant ESR1 circulating tumor DNA levels were observed. The median progression-free survival was 3.5 months. Treatment-related adverse events were mainly fatigue, hot flashes, and nausea.ARV-110 and ARV-471 are currently being further evaluated in clinical trials and have the potential to be approved.

**Figure 2:**
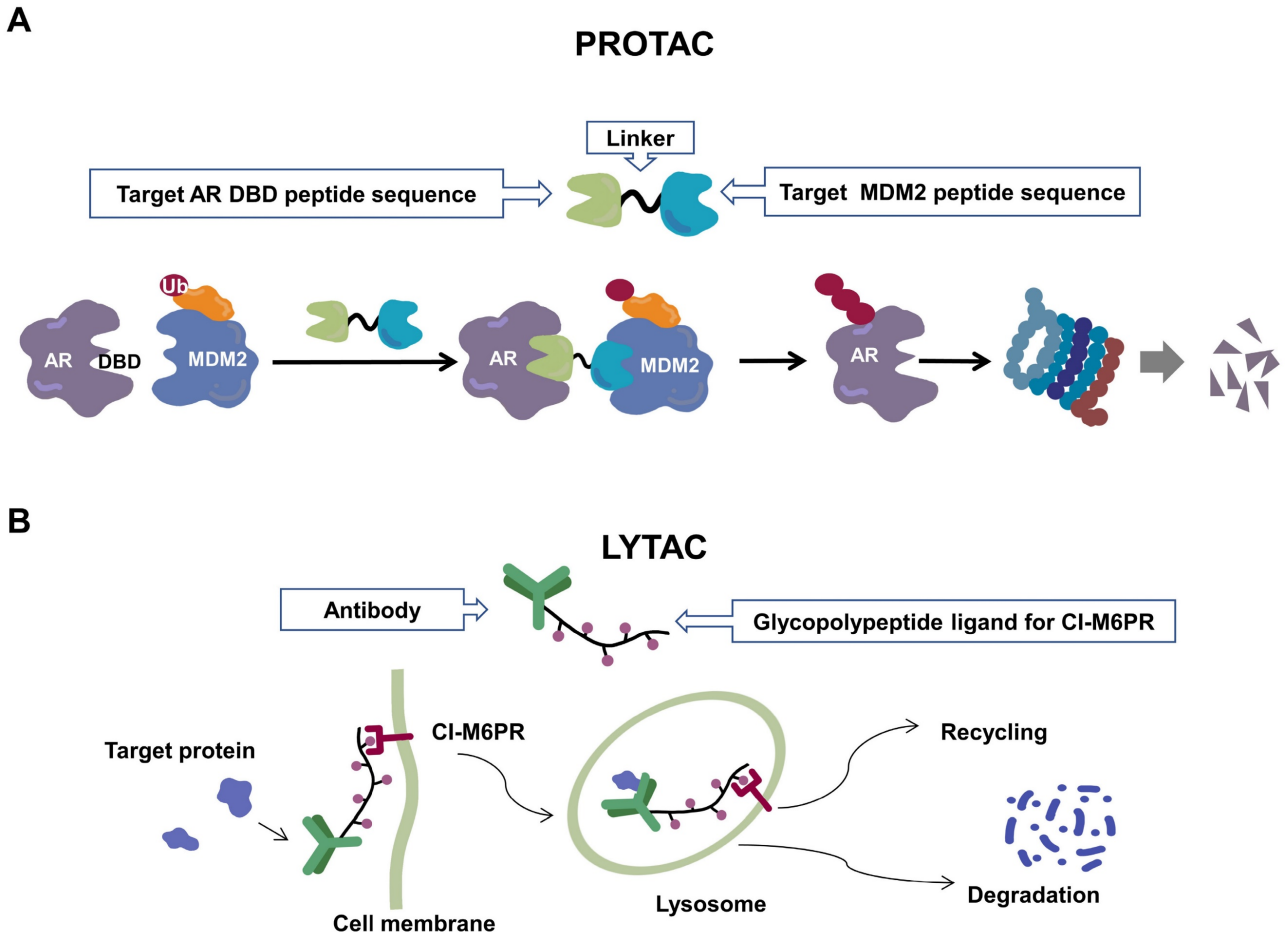
Schematic diagram of mechanism of PROTACs and Lysosome-Targeting Chimaeras (LYTACs). (A)An illustration of PROTAC (Au-AR pep-PROTAC) -mediated degradation of the AR and AR-V7. (B) An illustration of LYTAC-mediated degradation.

Recently, a group designed and synthesized Au-AR pep-PROTAC targeting AR-V7 by recruiting MDM2^172^. AR-V7 is often observed in CRPC. It is considered non-druggable due to the lack of ligand binding domain^120^, ^121^. In this study, they designed a novel peptide antagonist to target the DNA binding domain (DBD) of AR-V7 by artificial Inelligence (AI)-aided peptide drug design, which appears to have stronger affinity and specificity than small molecules. Due to the poor membrane permeability and short half-life of peptide PROTAC, an ultrasmall gold (Au)-peptide complex platform was developed for *in vivo* delivery of AR DBD PROTAC. It was shown that Au-AR pep-PROTAC induced degradation of AR and AR-V7 and inhibited cancer cell proliferation at both cellular and animal levels (Figure 2). KT-333 is a potent degradation agent that specifically degrades undruggable STAT3 in tumor cells. KT-333 causes a decrease in STAT3 levels* in vitro* and *in vivo* and induces tumor cell death^173^. KT-333 is in a Phase Ⅰ clinical trial (ClinicalTrials.gov Identifier: NCT05225584) to evaluate its safety, pharmacokinetics, pharmacodynamics, and clinical activity in adult patients with refractory lymphoma, large granular lymphocytic leukemia, solid tumors.

Three are still some challenges for us to apply PROTACs in clinic. First, the biological activity of PROTACs cannot be well predicted. Due to the different working principles, we cannot draw reliable conclusions based on the inhibitors of POI^174^. Second, the large molecular weight of PROTACs does not conform to Lipinski's Law of Five, which would affect their pharmacokinetic properties^175^. Thirdly, the degradation activity of PROTACs may be heterogeneous in different tissues or cells^176^ due to the different expression of E3 in different tissues or cells^177^. Fourth, acquired resistance to PROTACs may emerge^178^.

There are >600 E3 ubiquitin ligases in humans^179^ and only a few are currently used for PROTAC development, including MDM2, CRBN, and IAP1. As the functions and tissue-specific expression of other E3 ligases are understood, more new E3 ligases such as RNF4^180^, RNF114^181^, ^182^ and KEAP1[167]will be used for the development of PROTACs in future 176. A lot of effort and technology is required for the structural optimization of PROTACs drugs during the drug development process, which is costly and the results are very uncertain 183, 184. The development of new technologies, such as NanoBiT system^185^, enables the prediction and even real-time characterization of PROTACs-mediated degradation^186^.

### 3.2. Molecular glue

The concept of molecular glue was first introduced in the 1990s^187^. Molecular glue "glues" together molecules that would not normally bind together by modifying the surface of the protein. The formation of a ternary complex facilitates the dimerization or colocalization of two proteins, thereby modulating their function. Cyclosporin A and FK506 were the first examples of molecular glue, followed by the discovery of rapamycin^187^. Like PROTACs, molecular glue degraders utilize UPS for the degradation of POIs. Unlike PROTACs, they do not bind both E3 ligase and target protein but one of the two. The interaction of the three is further induced or stabilized, subsequently leading to target protein ubiquitination and proteasome degradation. Since molecular glues do not have linkers, they have a smaller molecular weight similar to small molecules and therefore have better cell permeability^188^. A compelling example of glue degraders is thalidomide-like immunomodulatory drugs (IMiDs). They can function as protein degraders by binding to CRBN. It has been shown that Ikaros family zinc finger protein 1 (IKZF1) and IKZF3 can be ubiquitinated and degraded by forming complexes with IMiDs and CRBN^189^. This would be beneficial for the treatment of multiple myeloma and 5q-deletion-associated myelodysplastic syndrome, respectively^190^, ^191^. Iberdomide (CC-220) is a novel IKZF1/3 degradant. It has shown meaningful clinical efficacy in clinical trials (ClinicalTrials.gov NCT02773030) of patients with relapsed or refractory multiple myeloma. In the dose-escalation cohort (n=90), the overall response rate was 32%. In the dose-expansion cohort (n=107), the overall response rate was 26%. The most common adverse events of grade 3 or worse were neutropenia, anemia, infection, and thrombocytopenia. There was one treatment-related death, and 5 patients (5%) discontinued treatment because of adverse events^192^. Therefore, further evaluation of its efficacy in the treatment of myeloma is needed. It's important to note that the discovery of molecular glues is usually serendipitous and achieving their rational design requires overcoming many challenges.

### 3.3 LYTACs

Since the UPS is located intracellularly, PROTACs target intracellular proteins. Unlike PROTACs, LYTACs^193^ mainly target extracellular and membrane-associated proteins, which comprise about 40% of the proteome and play key roles in disease progression^194^. LYTACs tagged with mannose-6-phosphonate link extracellular or membrane-bound proteins of interest to cation-independent mannose-6-phosphate receptors via the endosomal-lysosomal pathway to mediate protein endocytosis and lysosomal degradation^193^, ^195^. A recent study reported the development of molecular degraders of extracellular proteins through the asialoglycoprotein receptor that mediates the formation of ternary complexes through the same pathway, ultimately leading to endocytosis and degradation of target proteins 196 (Figure 2). Even though relatively few experimental studies have been conducted, the development of LYTACs has advanced therapeutic strategies targeting membrane and extracellular proteins.

Other protein degradation technologies include photodegradation-targeting chimeras^197^, macroautophagy degradation targeting chimeras^198^, autophagy-targeting chimera^199^, autophagy-tethering compounds^200^, and specific and non-genetic IAP-dependent protein erasers^201^. Targeted protein degradation strategies have gained considerable attention in the field of undruggable targets. Although these targeted degradation agents are reportedly effective, the results of experimental *in vivo* studies are insufficient and potential adverse events have not been addressed. In addition, the off-target toxicity, large molecular size, and high molecular complexity must be further optimized^202^, ^203^.

## 4. Targeting PPIs

A single protein is often insufficient for most biological processes, thus most are conducted *via* PPIs. To date, more than 14,000 PPIs have been identified in humans^204^. These protein complexes are involved in many critical cellular functions, including cell growth, DNA replication, transcriptional activation, translation, and transmembrane signaling. In the rapidly advancing field of target-based drug discovery, inhibitors of PPIs have received increased attention. However, PPIs are considered undruggable targets due to the structural characteristics of the complex interfaces, such as large and highly hydrophobic interfacial regions, flat interfaces that are difficult to bind to inhibitor molecules, and interfaces with amino acid residues that bind to each other with high affinity and are difficult to target with SMIs^205^.

### 4.1. SMIs

Accumulating evidence has challenged the traditional view that the structural characteristics of the PPI interface renders PPIs difficult to target with small-molecule drugs. The identification of “hot spots” has allowed the continued development of SMIs targeting PPIs^206^. Hot spots are key residues that are critical for high-affinity binding and significantly smaller in area than the interface. On average, hot spots account for 9.5% of interfacial residues^207^, ^208^. Competitive binding of a small-molecule ligand to a hot spot will block the original PPI^209^. Therefore, it is feasible to design SMIs to target the hot spots of PPIs. To date, several approaches have been employed for the development of SMIs targeting PPIs, including HTS, fragment screening, and virtual screening (VS). HTS, a major drug discovery paradigm, can rapidly and automatically screen hundreds of thousands of compounds for therapeutic applications over a relatively short period of time^210^. Applied techniques include fluorescence polarization-based drug screening^211^, small-molecule microarray drug-screening platforms^212^, surface plasmon resonance^213^, MALDI-TOF mass spectrometry^214^, and fluorescence resonance energy transfer (FRET)^215^. However, compound libraries are typically screened against traditional targets and, therefore, the rate of hits against targets of PPI is relatively low^216^, ^217^. Currently, the most successful examples of the application of HTS to identify targeted PPI inhibitors are the small molecules nutlins and benzodiazepinediones that target p53-MDM2 interactions^218^, ^219^. Fragment-based drug design (FBDD) is a method for developing effective compounds from fragments by random screening based on molecular structure^220^ using highly sensitive screening techniques, such as nuclear magnetic resonance (NMR)^221^ and X-ray crystallography^222^, to identify suitable low molecular weight fragments with low affinity, followed by structural optimization to produce drug-like molecules^223^. FBDD is reportedly more effective than HTS for discovery of PPI inhibitors 224. However, FBDD is limited by the difficulty in interpreting fragment hits to accurately identify drug candidates. The discovery of the novel antitumor drug ABT-263 (Navitoclax) 225, a Bcl-xL inhibitor, is considered a groundbreaking achievement for FBDD 226, 227. VS is a complementary technique to aid HTS in drug development and can be divided into ligand- and structure-based VS^224^, which has greater promise of success for PPI targets with well-defined hot spots. However, not all screened compounds can be successfully synthesized. In addition to the screening strategies described above, there are drug design strategies, including anchor-based PPI inhibitor design and design of small molecule mimics involved in PPI secondary structures. Anchors are hot residues of donor proteins and secondary structures that mediate most activities of PPIs^228^. Therefore, the use of small-molecule mimics of the key interactions of these components is an attractive strategy for modulation of PPIs^229^. The synthetic strategy is instead aimed at expanding the chemical space for screening of inhibitors^230^.

### 4.2. Peptide-based drug discovery

**Figure 3:**
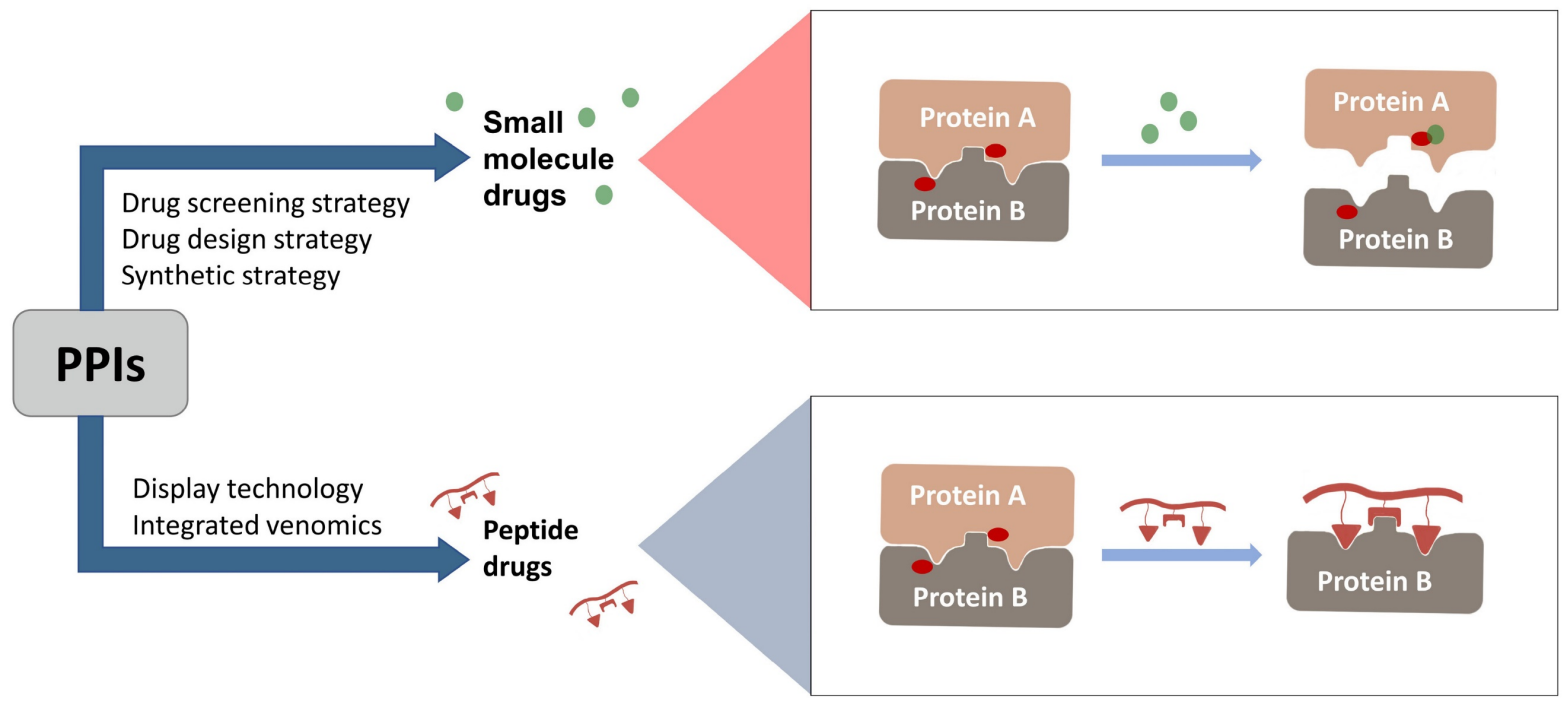
Orthosteric inhibition of PPIs by SMIs and peptide inhibitors. The red dots indicate “hot spots”.

In 2021, the number of peptides approved is at an all-time high^231^. Five peptides are approved for clinical use in 2022, representing 15% of the drugs approved by the FDA in 2022^4^. Peptide inhibitors are typically composed of less than 50 amino acids^232^ and inhibit protein binding by modifying important residues at the interface of the original PPIs, such as hot spots^233^. Peptidomimetics are defined as peptide-like molecules containing amino acid analogs and other chemical moieties with specific pharmacophores^234^ that bind competitively to PPI binding partners through similar structures, thereby blocking the original PPIs^235^. Peptides have higher selectivity and lower toxicity as compared to small-molecule drugs, and are cheaper to develop, more stable, and smaller with superior penetration than biologics^236^, ^237^. Therefore, peptides remain a promising class of drug candidates to target PPIs. Peptides have the combined advantages of small-molecule and protein drugs, but also some disadvantages, including low cell permeability, poor oral bioavailability, and short half-lives after administration, which pose great challenges for peptide-based drug discovery^237^, ^238^. Cyclization and modification of the backbone have emerged as two major strategies to address these issues^239^. General strategies for cyclization include hydrogen-bonded substitutes, stapling, and hairpins, which improve binding affinity, selectivity, and bioavailability by conformational restriction of the peptide^238^. A previous report proposed nucleophilic substitution of aromatic moieties for the synthesis of macrocyclic peptides by N-arylation^236^. This method was employed to obtain potent peptide inhibitors of p53-MDM2 with improved proteolytic stability and cell permeability^240^. Rational modification of the peptide backbone structure can reduces sensitivity to protein hydrolases and improve metabolic stability^239^. Display technologies, such as phage display, allow the creation of phage libraries through genetic modification for screening of peptide molecules that specifically bind to target molecules^241^, ^242^. In addition, integrated venomics allows bioinformatics analysis of genomic and transcriptomic data for screening of venom peptides^243^ as specific therapeutic targets for the development of peptide-based drugs for undruggable targets. To date, more than 60 peptide-based drugs targeting PPIs have been developed, optimized, and approved for clinical application^244^ (Figure 3).

MDM2 is a negative regulator of P53 and mediates its degradation by binding to p53^245^. Numerous studies have shown that blocking the p53-MDM2 interaction can restore the tumor suppressor function of P53^246^. The key interface residues involved Phe19, Trp23 and Leu26 on P53^247^. This is an important structural basis for inhibitor development. Since the discovery of Nutlin, several new potential inhibitors of the p53-MDM2 interaction have emerged, some of which have shown some positive results in preclinical and clinical studies. Based on a full understanding of the structure of MDM2, dihydroisoquinolinone was selected as the scaffold of inhibitor by VS. A combination of X-ray crystallography, molecular modeling, and iterative medicinal chemistry stepwise optimization eventually led to the discovery of NVP-CG1097^248^. NVP-CG1097 is a specific and highly selective p53-MDM2 inhibitor. In preclinical studies, it induced an increase in p53 expression^249^. However, NVP-CGM097 did not appear to exhibit significant tumor regression and had some undesired hematologic toxicity in the phase Ⅰ clinical trial^250^. But there is no denying that this is a very meaningful attempt. In addition to SMIs, current peptide-based PPI inhibitors are also a promising class of drug candidates. β-peptides^251^, peptoids^252^, N-acylpolyamine^253^ have previously been extensively studied to target the p53-MDM2 interaction. Currently, γ-AApeptide is receiving increasing attention as a novel peptidomimetic inhibitor that can disrupt the p53-MDM2 interaction^254^. To further enhance the inhibitory potency, sulfonamide groups were added to induce the scaffold to bend and fold^255^. Subsequently, from the addition of chiral side chains^255^, development of D-sulfono-γ-AApeptide^256^ to 1:1α/sulfono-γ-AApeptide^257^, which showed a gradual increase in inhibitory potency and resistance to hydrolysis. This suggests that rational design of peptidomimetics is a promising way to block the p53-MDM2 interaction.

## 5. Targeting IDPs

Intrinsically disordered regions (IDRs) and IDPs lack stable secondary and/or tertiary structures^258^, have low sequence complexity, lack large hydrophobic residues, are rich in charged and polar residues, and are highly flexible^259^. Over 30% of the eukaryotic proteins are IDPs and are involved in many important processes^260^. There is an association between dysregulation of IDPs and many human diseases, such as cancer, neurodegenerative diseases, and diabetes, among others 261. Both p53 and C-MYC possess IDRs, which are major impediments to targeting with drugs. Currently, there are three main strategies to target IDPs: (1) stabilize disordered states to prevent formation of toxic polymers; (2) inhibit interactions with other proteins to prevent formation of protein complexes with unfavorable biological functions; and (3) induce allosteric inhibition^262^. Since IDPs are widely involved with PPIs, such as p53-MDM2 and C-MYC-MAX, the development of drugs to target IDPs involved in protein complexes employs some of the same strategies for targeting PPIs, such as FBDD. Commonly used drug discovery techniques include NMR, small-angle X-ray scattering, circular dichroism, FRET, and simulations of molecular dynamics (MDs). Among them, NMR is used to study the transient interactions of ligands with proteins and is arguably the best technique to observe and characterize the structural dynamics of IDRs^263^, while small-angle X-ray scattering and FRET are highly complementary to NMR^264^. With different advantages and disadvantages, NMR revealed more detailed structural dynamics of C-MYC-MAX and smaller molecular weights of rigid proteins. For C-MYC-MAX, three NMR structures (PDB codes: 1A93, 1MV0, and 2A93) were identified^265^, ^266^. Although NMR provides snapshots of dynamic structures (20 models of 1MVO and 40 models of 2A93), the lengths of the three structures were less than 30 amino acids. For the X-ray determined structures of C-MYC-MAX (PDB codes: 1NKP, 6G6J, 6G6K, and 6G6L), the lengths of the structures were greater than 80 amino acids. In addition, the interactions between C-MYC-MAX and DNA (5^'^-CACGTG-3^'^) were clearly revealed^267^, ^268^. The high-resolution structure of 1NKP explained how bHLHZ heterodimers can mediate specific and high-affinity binding to DNA, throwing light on the drug design targeting the C-MYC-MAX complex.

In contrast to the conformation-based approaches described above, the MD-based approach using MD simulations of the behavior of IDPs under physiological conditions in a solvent provide greater insight into protein dynamics^269^. The structure-based strategy was utilized to design efficient peptides to disrupt p53-MDM2/X interactions. In a prior study, the critical residues of validated inhibitors of MDM2/X were analyzed and computational HTS as well as MD simulations were conducted of two mutants, pDI (LTFEHYWAQLTS) and pDIQ (ETFEHWWSQLLS), which provided theoretical structural information of 27 native and mutants of p53-based inhibitory peptides^270^. A new Bayesian inference approach (MELD × MD) was used to simulate five peptides binding to MDM2, including the p53 epitope, pdiq, ATSP-7041, and two negative alanine-based peptides, which depicted the most likely bound conformations of MDM2 ligands^271^. Furthermore, physics-based atomic simulations can effectively overcome the uncertainties of disordered ensemble calculations and are expected to provide the rigorous thermodynamic ensembles required to reliably describe IDP-ligand interactions^264^. Even though several experimental and computational studies have offered insight into IDPs, additional experimental techniques are needed for drug discovery. Further understanding of the structure and function of IDPs is particularly important for treatment of neurodegenerative diseases associated with abnormal peptide aggregation. Moreover, IDPs are associated with the regulation of liquid-liquid phase separations in cells, which drives the formation of membrane-free cell organelles and the localization of biomolecules 272, 273, and may provide a novel approach to elucidate the roles of IDPs in tumors and neurological diseases.

## 6. Targeting protein-DNA binding

TFs are the most widely studied drug targets to block gene expression for cancer treatment. Although blocking protein-DNA interactions appears to be a more direct approach, studies have been limited. It is difficult to develop specific SMIs due to the large interface area of protein-DNA interactions, the large number of anchor points, the highly positive charge, and the fact that the DNA binding sites of related proteins are usually highly conserved 274, 275. Despite these problems, the remarkable advances in CADD and experimental techniques have facilitated the development of protein-DNA inhibitors. Deep docking, an ultra-fast AI-based method, can significantly improve the efficiency of drug screening with significant runtime savings while handling large chemical libraries^276^. In addition, the most commonly used databases for AI-based prediction of binding affinity currently include the PDBbind database, Comparative Assessment of Scoring Functions benchmark dataset, and BindingDB, a web-accessible database of measured binding affinities 277-279. The continued development of AI and deep learning has provided greater possibilities for protein structure prediction and structure-based drug design. Existing machine learning methods for prediction of binding sites can be divided into classical non-deep learning methods and modern deep learning methods, such as P2Rank 280 and DeepSurf 281. In general, several computational tools, such as homology modeling^282^ and ProDFace^282^, can be used to predict druggable sites on the surface of DNA-binding domains, which are highly conserved and not prone to mutations, providing opportunities for drug development^283^. The DNA-binding interface of the AR was successfully identified using homology modeling and VS 284, 285. InS3-54 is a STAT3-DNA binding inhibitor identified using VS and screened from 2 million compounds. InS3-54 has an IC50 of 13.8 ± 0.4 μM and binds selectively to STAT3. The main residues with which STAT3 interacts are Met331, Val343, Ile467, Met470, Lys 340 and Asn466. And InS3-54 does not bind to STAT1 due to the conflict of residues Pro326 and Thr327 with InS-54. In several lung and breast cancer cell lines, InS3-54 induced apoptosis to inhibit cancer cell growth and inhibited cancer cell migration and invasion^286^. Hence, CADD greatly improves the feasibility of targeting protein-DNA sites.

## 7. Summary

This review provides an overview of the current status of undruggable targets and potential solutions from several perspectives. Bifunctional small molecules enable targeted degradation of proteins of interest through various endogenous pathways, which can reduce the overall levels of disease-associated proteins and broaden the development of drugs for specific targets. At least 20 PROTACs against the targets mentioned above have been developed. Various drug entities and discovery techniques have its limitations. The permeability and bioavailability are poor due to its large molecular weight, and the degradation activity of target protein is difficult to evaluate. Also, the drug properties, toxicities and resistance of degraders need to be validated by adequate experiments. The stability of targeting PPI drugs like peptide should be further improved. High-throughput screening need to expand their screening libraries to improve hit rates, and the challenge for FBDD and virtual screening is to successfully translate screening hits into drug candidates.

Emerging technologies like excellent drug delivery system, conditional activated PROTACs (light-induced protein degradation), tissue specific E3 ligases and its ligands, high throughput screening method of active PROTACs may be helpful to overcome PROTACs limitations in the future. AI is also very helpful to discover and develop new target protein, obviously, which can also shed some lights into the overcome of the undruggable targets. AI technologies, such as DeepChem, DeepTox, and DeepNeuralNetQSAR, have been applied in virtual screening to predict the physicochemical properties, biological activities, and toxicities of drug molecules. AI can also help to predict the three-dimensional structures of target proteins and drug-protein interactions. AlphaFold significantly outperformed other protein structure prediction methods. This will help researchers better understand protein structures and interactions, which in turn will speed up the process of drug development. For example, using Alphafold's prediction results, candidate drug molecules that interact with target proteins can be quickly screened and their structures optimized for improved affinity and specificity. Alphafold also has some limitations, such as not being able to solve the long-standing protein folding problem; predicting a single ranked structure of a protein sequence, which cannot directly address the allosteric mechanism; and being less applicable to IDPs and IDRs, using low structural probabilities to describe them^287^, ^288^. Therefore, a combination of approaches is needed to address these targets. Moreover, further discovery of new drug targets, such as some proteins involved in cell metabolism, cell death pathways, seems to be beneficial for the treatment of diseases. Another approach is to develop activators of proteins against some targets, such as tumor suppressors activators, which seems to be another concise approach.

With the help of new drug-development strategies, some undruggable target proteins like RAS, HIF-2α, BCL-2, MDM2, and MLL are no longer considered undruggable right now. It remains a huge challenge to completely overcome the undruggable proteins, and new drug development strategies, better extraction processes that do not disrupt protein-protein interactions, and more precise AI are urgently needed. In any case, we believed that it is only a matter of time before undruggable targets become druggable.

## Figures and Tables

**Table 1:** Ongoing clinical trials of KRAS^G12C^-targeted inhibitors and related combination therapy clinical trials.

Drug	Treatment Strategy	ClinicalTrials.gov registration	Disease setting	Study phase	Recruitment Status
AMG510 (Sotorasib)	Monotherapy	NCT03600883	Advanced solid tumors with KRAS G12C mutations	I/II	Active, not recruiting
	Monotherapy	NCT04667234	Advanced/unresectable/metastatic NSCLC with KRASG12C mutation	/	Available
	Monotherapy	NCT04380753	Advanced/Metastatic Solid Tumors with KRASG12C Mutations	I	Active, not recruiting
	Monotherapy	NCT04625647	Advanced NSCLC with KRAS G12C mutations	II	Recruiting
	Monotherapy	NCT04933695	Stage IV NSCLC with KRAS G12C mutations without prior treatment	II	Active, not recruiting
	Monotherapy	NCT05400577	Stage IV NSCLC with KRAS G12C mutations without prior treatment	II	Recruiting
	Monotherapy	NCT05398094	Stage III unresectable NSCLC with KRAS G12C mutations	II	Recruiting
	Monotherapy	NCT05273047	Metastatic NSCLC with KRAS G12C mutations	/	Recruiting
	Monotherapy	NCT05451056	NSCLC with KRAS G12C mutations	II	Not yet recruiting
	Monotherapy vs. Docetaxel	NCT04303780	Advanced NSCLC with KRAS G12C mutations	Ⅲ	Active, not recruiting
	Combined with Tarloxotinib	NCT05313009	NSCLC with KRAS G12C mutations	I/II	Recruiting
	Combined with BBP-398	NCT05480865	Advanced solid tumors with KRAS G12C mutations	I	Recruiting
	Combined with VS-6766	NCT05074810	NSCLC with KRAS G12C mutations	I/II	Recruiting
	Combined with targeted therapy, chemotherapy, or immunotherapy	NCT04185883	Advanced Solid tumors with KRAS G12C mutations	I/II	Recruiting
	Combined with Panitumumab vs. Trifluridine and Tipiracil,or Regorafenib	NCT05198934	CRC with KRAS G12C mutations	Ⅲ	Active, not recruiting
	Combined with MVASI	NCT05180422	Advanced, unresectable or metastatic KRASG12C mutant NSCLC With asymptomatic brain metastasis	I/II	Recruiting
	Combined with Panitumumab	NCT05638295	Advanced/Metastatic Solid Tumors With KRASG12C Mutation	II	Not yet recruiting
	Combined with RMC-4630	NCT05054725	NSCLC with KRAS G12C mutations	II	Recruiting
	Combined with cisplatin or carboplatin and pemetrexed	NCT05118854	Stage IIA-IIIB resectable non-squamous NSCLC with KRAS G12C mutations	II	Recruiting
MRTX849 (Adagrasib)	Monotherapy	NCT05162443	Advanced solid tumors with KRAS G12C mutations	/	Available
	Monotherapy	NCT05263986	Advanced/Metastatic Solid Tumors With KRASG12C Mutations	I	Active, not recruiting
	Monotherapy	NCT05634525	Metastatic pancreatic cancer with KRAS mutations	I	Not yet recruiting
	Monotherapy	NCT05673187	Stage IV NSCLC with KRAS G12C mutations	II	Not yet recruiting
	Monotherapy or combined with Nivolumab	NCT05472623	Resectable NSCLC with KRAS G12C mutations	II	Not yet recruiting
	Monotherapy or combined with Cetuximab or Pembrolizumab or Afatinib	NCT03785249	Advanced solid tumors with KRAS G12C mutations	I/II	Recruiting
	Monotherapy or combined with Pembrolizumab	NCT04613596	Advanced or metastatic NSCLC with KRAS G12C mutations	II/Ⅲ	Recruiting
	Monotherapy vs. Docetaxel	NCT04685135	Advanced or metastatic NSCLC with KRAS G12C mutations	Ⅲ	Recruiting
	Combined with Cetuximab vs. Chemotherapy	NCT04793958	Advanced CRC with KRAS G12C mutations	Ⅲ	Recruiting
	Combined with BI 1701963	NCT04975256	Advanced solid tumors with KRAS G12C mutations	I	Completed
	Combined with Palbociclib	NCT05178888	Advanced solid tumors with KRAS G12C mutations	I	Active, not recruiting
	Combined with TNO155	NCT04330664	Advanced solid tumors with KRAS G12C mutations	I/II	Active, not recruiting
	Combined with SAR442720	NCT04418661	NSCLC with KRAS G12C mutations	/	Active, not recruiting
	Combined with Pembrolizumab	NCT05609578	Advanced NSCLC with KRAS G12C mutations	II	Recruiting
	Combined with Cetuximab and Irinotecan	NCT05722327	CRC with KRAS G12C mutations	I	Not yet recruiting
	Combined with VS-6766	NCT05375994	NSCLC with KRAS G12C mutations	I/II	Recruiting
JAB-21822	Monotherapy	NCT05009329	Advanced solid tumors with KRAS G12C mutations	I/II	Recruiting
	Monotherapy	NCT05276726	Advanced or metastatic NSCLC with KRAS G12C mutations	I/II	Recruiting
	Monotherapy or combined with Cetuximab	NCT05002270	Advanced solid tumors with KRAS G12C mutations	I/II	Recruiting
	Combined with JAB-3312	NCT05288205	Advanced solid tumors with KRAS G12C mutations	I/II	Recruiting
	Combined with Cetuximab	NCT05194995	Advanced solid tumors with KRAS G12C mutations	I/II	Recruiting
JDQ443	Monotherapy	NCT05329623	Small Cell Lung Carcinoma	I	Suspended
	Monotherapy	NCT05445843	Advanced or metastatic NSCLC with KRAS G12C mutations	II	Recruiting
	Monotherapy	NCT05132075	Advanced NSCLC with KRAS G12C mutations	Ⅲ	Recruiting
	Monotherapy or combined with TNO155 or tislelizumab or TNO155 + tislelizumab	NCT04699188	Advanced solid tumors with KRAS G12C mutations	I/II	Recruiting
	Combined with Trametinib or Ribociclib or cetuximab	NCT05358249	Advanced solid tumors with KRAS G12C mutations	I/II	Recruiting
GDC-6036	Monotherapy vs. Docetaxel	NCT03178552	Advanced/unresectable/metastatic NSCLC	II/Ⅲ	Recruiting
	Monotherapy or combined with chemotherapy, immunotherapy, etc.	NCT04449874	Advanced or metastatic NSCLC with KRAS G12C mutations	I	Recruiting
	Combined with Pembrolizumab	NCT05789082	Advanced or metastatic NSCLC with KRAS G12C mutations	I/II	Not yet recruiting
	Combined with Cetuximab	NCT04929223	Metastatic CRC	II/Ⅲ	Recruiting
D-1553	Monotherapy	NCT05383898	Advanced or metastatic NSCLC with KRAS G12C mutations	I/II	Recruiting
	Monotherapy or combined with other therapies	NCT04585035	Advanced or metastatic NSCLC with KRAS G12C mutations	I/II	Recruiting
	Combined with immunotherapy or targeted therapy	NCT05492045	Advanced or metastatic NSCLC with KRAS G12C mutations	I/II	Not yet recruiting
	Combined with IN10018	NCT05379946	Advanced or metastatic NSCLC with KRAS G13C mutations	I/II	Not yet recruiting
D3S-001	Monotherapy	NCT05410145	Advanced solid tumors with KRAS G12C mutations	I	Recruiting
GFH925	Monotherapy	NCT05005234	Advanced solid tumors with KRAS G12C mutations	I/II	Recruiting
YL-15293	Monotherapy	NCT05119933	Advanced solid tumors with KRAS G12C mutations	I/II	Recruiting
JNJ-74699157	Monotherapy	NCT04006301	Advanced solid tumors with KRAS G12C mutations	I	Completed
RMC-6291	Monotherapy	NCT05462717	Advanced solid tumors with KRAS G12C mutations	I	Recruiting
HS-10370	Monotherapy	NCT05367778	Advanced solid tumors with KRAS G13C mutations	I/II	Not yet recruiting
MK-1084	Monotherapy or combined with Pembrolizumab	NCT05067283	Advanced solid tumors with KRAS G14C mutations	I	Recruiting
LY3537982	Monotherapy or combined with targeted therapy, immunotherapy, etc.	NCT04956640	Advanced solid tumors with KRAS G12C mutations	I	Recruiting

**Table 2:** Physiological functions and causes of abnormal expression patterns of potential protein targets in various diseases.

Target protein	Physiological function	Abnormal expression	Disease
KRAS	Signal transduction	Mutation	Pancreatic cancer, colorectal cancer, non-small cell lung cancer, etc.
p53	Transcription	Mutation	Lung cancer, stomach cancer, liver cancer, etc.
C-MYC	Transcription	Overexpression	Lung cancer, stomach cancer, breast cancer, etc.
STAT3	Transcription	Overexpression	Rectal cancer, lung cancer, breast cancer, etc.
AR-V7	Transcription	Overexpression	Metastatic castration-resistant prostate cancer
β-catenin	Transcription	Abnormal accumulation	Colon cancer, hepatocellular carcinoma, pancreatic cancer, etc.
HOXA9	Transcription	Overexpression	Acute myeloid leukemia
MEIS1	Transcription	Overexpression	Acute myeloid leukemia
EWS-FLI1	/	Fusion	Ewing sarcoma
PTP	Post-translation modification	Elevated/reduced expression	Breast cancer, stomach cancer, prostate cancer, rheumatoid arthritis, systemic lupus erythematosus, etc.
HAT	Post-translation modification	Mutation	Leukemogenesis, Rubinstein-Taybi syndrome, etc.
SMARCA2	Chromatin remodelling	/	Non-small cell lung cancer, etc.

**Table 3:** The PROTACs developed for the undruggable targets in cancers.

Targets	PROTACS	Disease	Structure	Reference
KRAS	YF135	Non-small cell lung cancer	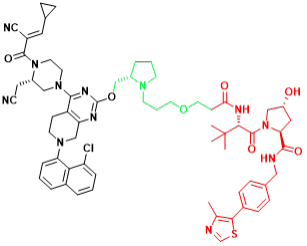	289
KRAS	KP-14	Non-small cell lung cancer	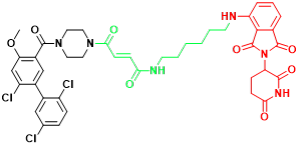	290
KRAS	LC-2	Lung cancer, Pancreatic cancer	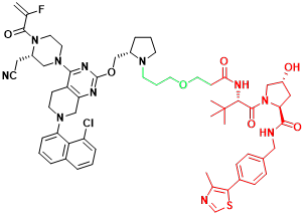	41
SHP2	ZB-S-29	Monocytic leukemia	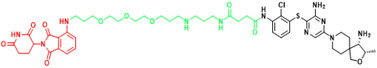	291
SHP2	SHP2-D26	Esophageal cancer, Monocytic leukemia	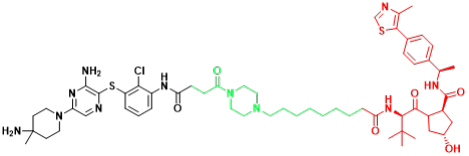	292
SHP2	SP4	Cervical cancer	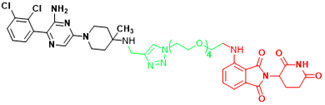	293
SHP2	R1-5C	Monocytic leukemia, Esophageal cancer, Acute myelogenous leukemia, Acute lymphoblastic leukemia	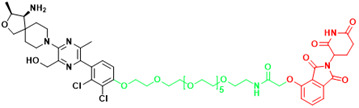	294
BRD4(C-MYC)	ARV-825	Burkitt's Lymphoma, B cell lymphoma	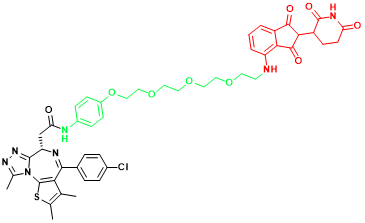	165
STAT3	SD-36	Acute myelogenous leukemia, Anaplastic large cell lymphoma	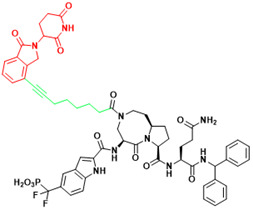	114
STAT3	SD-91	Acute myelogenous leukemia	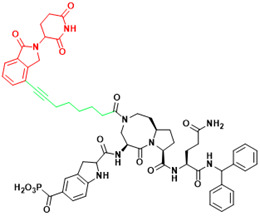	295
AR-V7	MTX-23	Castration-resistant prostate cancer	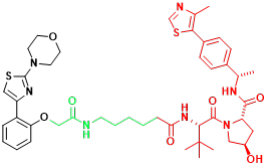	296
AR-V7	6	Castration-resistant prostate cancer	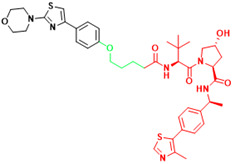	297
β-catenin	xStAx-VHLL	Rectal cancer	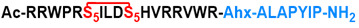	298
p300/CBP	dCBP-1	Multiple myeloma	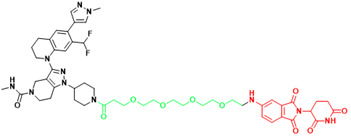	299
EP300	JQAD1	Neuroblastoma	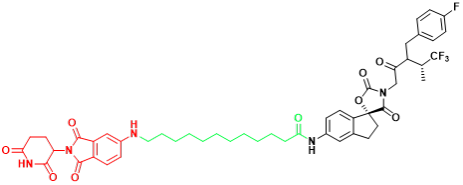	300
SMARCA2	A947	Non-small cell lung cancer	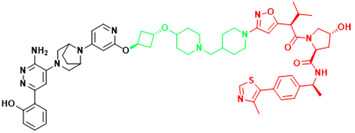	301
